# SAR validation of RF coils and implants in a B0‐free RF safety laboratory

**DOI:** 10.1002/mp.70636

**Published:** 2026-08-18

**Authors:** Nur Izzati Huda Zulkarnain, Mazin M. Mustafa, Alireza Sadeghi‐Tarakameh, Mert Ates, Jeromy Thotland, Steve Jungst, Arcan Erturk, Lance DelaBarre, Matt Waks, Russell L. Lagore, Kamil Ugurbil, Gregory J. Metzger, Gregor Adriany, Yigitcan Eryaman

**Affiliations:** ^1^ Center for Magnetic Resonance Research University of Minnesota Minneapolis Minnesota USA

**Keywords:** implants, MRI, radio‐frequency heating, RF coils, safety

## Abstract

**Background:**

As MRI continues to advance toward higher field strengths, RF safety assessment has become more complex. Accurate estimation of specific absorption rate (SAR) is essential for evaluating both hardware and implant safety, yet thermometry and simulation‐only approaches face limitations. In addition, these evaluations are typically performed within the MR scanner, where failures or excessive loading can damage the transmit chain and result in costly repairs and scanner downtime. To overcome these challenges, alternative experimental approaches that enable direct and controlled SAR validation outside the MR environment are needed.

**Purpose:**

This study demonstrates direct SAR validation of RF hardware and implants in a dedicated B0‐free RF safety laboratory. The work aims to establish direct SAR measurement as a practical and accurate alternative for quantitative safety verification while expanding the range of tools available for MR hardware evaluation beyond the constraints of the scanner environment.

**Methods:**

Hardware assessments were conducted in the safety lab equipped with a 16‐channel broadband RF amplifier system (45–450 MHz), a 100 dB shielded Faraday cage, automated 3D and 1D motion systems, and a high‐precision SAR probe. For coil validation, direct SAR mapping was performed on a custom‐built parallel transmit (pTx) coil and a commercial single‐channel RF coil at 447 and 297 MHz, respectively, and results were compared with electromagnetic simulations. Transfer function (TF) validation for a directional DBS electrode at 128 MHz was performed in a rectangular electric‐field generator, whose field distribution was first validated using the SAR probe. The same probe was then used to compare measured SAR with TF‐based SAR predictions, providing a direct quantitative validation of the TF method.

**Results:**

Measured SAR distributions showed strong quantitative agreement with simulations across all experiments. For the 8‐channel pTx head coil at 447 MHz, the normalized root‐mean‐square error (NRMSE) across four excitation patterns was below 13%. For the commercial single‐channel head coil at 297 MHz, plane‐wise comparisons across axial, coronal, and sagittal slices yielded an average NRMSE value of 7.18%, demonstrating spatial consistency between measured and simulated fields. The electric‐field generator used for DBS transfer function (TF) validation exhibited 3.68% NRMSE relative to simulation, confirming accurate field reproduction. SAR measurements using the same probe further showed close agreement with TF‐based SAR predictions across all seven lead trajectories, confirming both the accuracy and broadband applicability of the direct SAR method.

**Conclusion:**

Direct SAR measurement provides a reliable and quantitative approach for validating RF coil and implant safety, matching the accuracy of conventional scanner‐based thermometry and simulation methods. Conducting these measurements in a B0‐free RF safety laboratory further simplifies and accelerates the process by eliminating magnetic field constraints and the waiting periods required for the setup to return to thermal equilibrium in thermometric techniques. This configuration provides an efficient and controlled platform for systematic SAR validation across a wide range of MR frequencies.

## 1 INTRODUCTION

Ultra‐high field (UHF) MRI offers increased signal‐to‐noise ratio (SNR) and enhanced contrast.^1^, ^2^ The FDA approval of 7T MR scanners for clinical applications highlights the growing role of UHF MRI in healthcare. Meanwhile, imaging at even higher field strengths continues to be investigated. The 10.5 T system at the University of Minnesota as well as the 11.7 T system at NeuroSpin Centre at CEA Saclay have already produced their first in‐vivo human images.^3^, ^4^, ^5^, ^6^, ^7^ Additionally, researchers are preparing an additional 11.7 T system for human head imaging at the US National Institutes of Health and South Korea, while Germany, Netherlands and China are considering building 14.0 T human MRI scanners in the near future.^8^, ^9^, ^10^

Similar to their lower‐field counterparts, UHF MRI systems consist of several critical components including the magnet, which generates the static magnetic field; the gradient coils which allows spatial encoding of MR signal essential for image generation; and the RF coils which transmit/receive RF fields into/from the body for spin excitation/signal acquisition.^11^ While the magnet and the gradient coils typically use standardized technologies with minimal application‐specific modifications, RF coils are built in a variety of shapes and sizes depending on specific imaging applications.^12^, ^13^ When commercial UHF RF coil options are limited or unavailable, in‐house custom coil development becomes necessary. To address this need, researchers must design, build, and validate their own RF coils to meet specific imaging needs.^14^, ^15^, ^16^

As field strengths increase, ensuring patient safety becomes more challenging, primarily due to increasingly complex RF field interactions at higher frequencies.^17^ A critical safety concern in UHF MRI is the specific absorption rate (SAR), which increases with the field strength.^18^, ^19^, ^20^ Therefore, prior to human imaging, RF coils must undergo a rigorous safety validation process to establish safe operating conditions, including SAR limits that must be strictly adhered to at all times.^21^, ^22^ RF safety validation of a custom‐built RF coil is a complex procedure that involves demonstrating agreement between electromagnetic (EM) simulations and experimental measurements, including but not limited to transmit magnetic field (B1+) and local SAR distributions.^4^, ^22^, ^23^, ^24^, ^25^ Additional complexities emerge when metallic implants are present. RF‐induced currents on metallic implants can lead to significant heating in the surrounding tissues regardless of the B0 field strength.^26^ These localized SAR effects are not fully captured by standard coil characterization and require a thorough investigation due to stricter SAR limits imposed when imaging patients with implants. Together, these challenges highlight the critical importance of systematic and accurate RF safety assessments, both for RF coil validation and for evaluation of implant safety in MRI.

Traditionally, RF coil and implant safety validation studies have been conducted in the MRI scanner.^21^, ^23^, ^26^, ^27^, ^28^ However, this approach presents significant disadvantages, including high operational costs and limited scanner availability. For instance, in the US, one hour of experimental investigation on a conventional MR system can cost the researchers several hundred dollars, with costs being even higher for UHF MR scanners. Additionally, RF coil safety tests often rely on RF heating experiments (i.e., MR thermometry) using phantoms, where temperature increases are measured to estimate local SAR.^26^, ^28^, ^29^, ^30^, ^31^ These experiments are time‐consuming due to the prolonged wait times needed for the phantom to reach thermal steady‐state between experiments.^32^, ^33^ The challenge is exacerbated in parallel transmit (pTx) arrays, where multiple excitation scenarios must be tested individually due to their unique SAR patterns, often extending the experimental durations to several days. Beyond duration, MRT‐based tests face additional technical challenges, including phase drift errors, system instabilities, and the need for specialized phantoms. Moreover, they typically require high transmit power to generate measurable temperature changes, posing a risk to sensitive receiver electronics, particularly when a dedicated receive array is included within the transmit coil.

Given these limitations, direct SAR measurement is a practical alternative to thermometry and simulation‐based validation for assessing RF safety. Here, validation refers to experimentally evaluating electromagnetic models and simulations of RF coils and implants by comparing simulated and measured SAR under controlled conditions in the RF safety lab. In this study, a high‐precision SAR probe was used to compare the SAR of a custom‐built pTx coil and a commercial single‐channel RF coil operating at 447 and 297 MHz against simulations. The same probe was used to validate the transfer function of a directional deep brain stimulation (DBS) electrode at 128 MHz. All measurements were conducted in a B0 field‐free RF safety lab which provides a controlled environment that eliminates the risks associated with scanner‐based testing. Together, this setup provides an efficient and systematic framework for RF safety assessment of RF coils and implants.

## 2 METHODS

### 2.1 Facility

**FIGURE 1:**
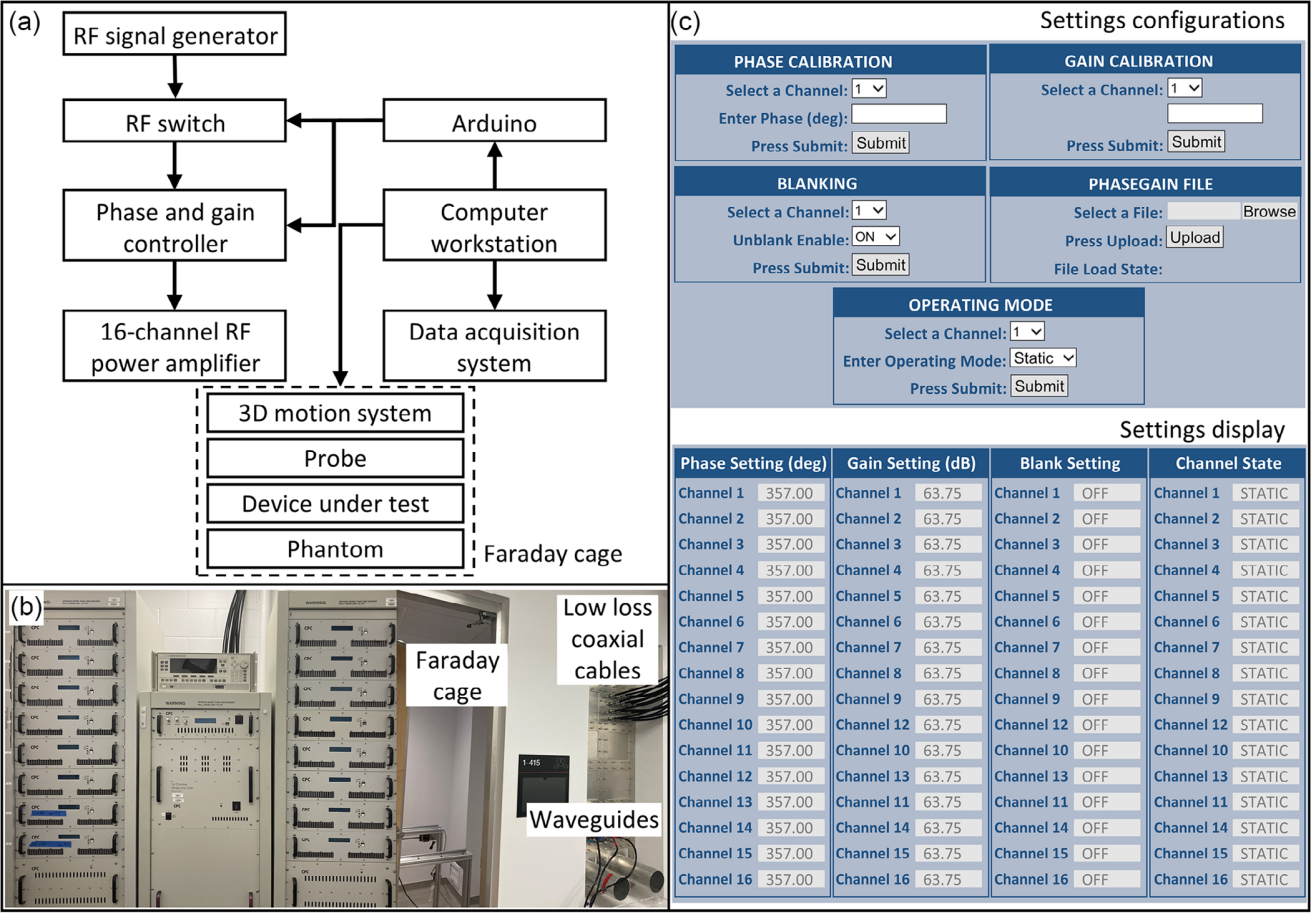
Configuration of the RF safety lab for controlled RF safety testing. (a) Schematic layout and operational workflow of the RF safety lab. (b) A 16‐channel broadband RF transmit system delivers customizable excitation patterns via low‐loss coaxial cables. Fiber optic cables for data measurements are routed through waveguides. (c) A user interface for controlling amplitude and phase for each RF channel.

The RF safety lab is a dedicated facility within the Center for Magnetic Resonance Research (CMRR), equipped with a 16‐channel broadband power amplifier system for RF excitation and a Faraday cage for EM interference shielding.^34^ It also features a dosimetric probe for SAR validation studies and automated 3D and 1D linear motion systems for precise probe positioning.^35^, ^36^, ^37^ A centralized computer hosts the user interface for controlling the RF power amplifier system, linear motion systems, and data acquisition tools that cater to a wide range of safety validation experiments. Figure 1a shows the schematic layout and operational configuration of the safety lab.

The custom‐built RF amplifier system used in the safety validation studies is similar to the system described in a previous work,^36^ as shown in Figure 1b. An RF signal generator (Hewlett‐Packard 83620B‐H80, Palo Alto, CA) drives a phase and gain controller (PGC) (Communications Power Corporation (CPC), Hauppauge, NY), which independently modulates amplitude and phase for each channel. The output channels are connected to broadband RF amplifiers (45‐450 MHz CPC MRI Plus), each capable of delivering a peak power of 2 kW. A microcontroller (Arduino Uno, Scarmagno, Italy) provides an un‐blanking signal to the PGC unit and controls the RF pulse duration and duty cycle during excitation. All studies were conducted inside a Faraday cage with dimensions of 2.6 × 2.1 × 2.6 m^3^ and up to 100 dB isolation to prevent RF interference with ongoing MRI studies at the CMRR. RF power from the amplifiers is routed into the cage using LMR‐400 coaxial cables through a panel‐mounted interface, while fiber optic probe cables are passed through dedicated waveguides to maintain shielding integrity.

To calibrate the transmit system, the amplifier outputs are connected to 50 dB directional couplers (Model C8212‐13, Werlatone, Inc., Patterson, NY). The attenuated forward signals are monitored via an oscilloscope to measure and adjust amplitude and phase across all channels. Phase and gain settings are modified on the computer workstation to generate the desired RF excitation patterns through a user interface as shown in Figure 1c. Once the calibration is complete, the directional couplers are removed, and the amplifier outputs are directly connected to the RF coil or resonator elements using the same set of cables employed during calibration. After calibration, the cables are connected to an RF coil. In certain circumstances, continuous monitoring via oscilloscope is also possible during RF power delivery.

For accurate probe positioning with submillimeter accuracy, a computer‐controlled 3D positioning system (COSI Measure) is used and placed inside the Faraday cage.^35^ The positioning system is equipped with three stepper motors, allowing precise data acquisition along three orthogonal axes with a working volume of 500 × 500 × 700 mm^3^. Outside the Faraday cage, a 1D linear motion system was used to measure the transfer function of elongated metallic implants.^37^ The motorized linear guide has a travel length of 100 cm and is mounted on an acrylic container measuring 12 × 12 × 120 cm^3^, which is filled with tissue‐mimicking medium.

### 2.2 RF coil characterization via SAR validation at 447 and 297 MHz

Coil validation experiments were conducted to compare simulated and measured specific absorption rate (SAR) distributions for two RF transmit coils: a custom‐built 8‐channel transmit/receive bumped dipole head coil at 447 MHz,^4^ and a commercial single‐transmit (1Tx) head coil (Nova Medical, Wilmington, MA) operating at 297 MHz. Detailed descriptions of the simulation models for both coils were previously reported by Sadeghi‐Tarakameh et al and Mustafa et al.^4^, ^38^ Direct SAR mapping was performed using COSI Measure system described in Section 2.1, along with a high‐precision, isotropic E‐field dosimetric probe (EX3DV4, SPEAG, Zurich, Switzerland).

All experiments were conducted using an open‐top cylindrical phantom (70 mm radius, 190 mm height) filled with a polyvinylpyrrolidone (PVP) solution, positioned at the center of the coil cavity.^39^ The dielectric properties of the PVP phantom were matched to the SAR probe's calibration conditions at the corresponding operating frequencies The dielectric properties of the PVP phantom were matched to the SAR probe's calibration conditions at each operating frequency (σ = 0.90 S/m, ε = 55 at 300 MHz; and σ = 0.97 S/m, ε = 53 at 450 MHz). As part of system validation, SAR linearity with respect to input RF power was evaluated at two fixed locations using the circularly‐polarized (CP) mode of the bumped dipole coil, one at the center and another near the periphery of the phantom.

**FIGURE 2:**
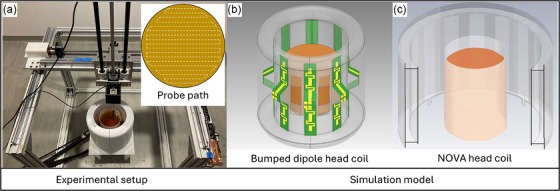
Experimental and simulation setup for coil characterization at 447 and 297 MHz. (a) 3D SAR measurement system with a high‐precision dosimetric probe for volumetric mapping. Probe trajectory covering the circular surface area of the phantom is shown. (b) Simulation of the bumped dipole head coil with comparison of 10 g‐averaged SAR in an axial plane near the excitation port for different excitation patterns. (c) Simulation of the NOVA head coil with comparison of 10 g‐averaged SAR in center axial, coronal, and sagittal planes.

For volumetric acquisition, the 3D motion system guided the SAR probe along a predefined path programmed using motion control software (Mach3, Newfangled Solutions, ME). The probe advanced in 7 mm isotropic steps with temporal averaging at each dwell point and recorded measurements at a temporal resolution of 0.25 s. Probe motion and acquisition were carefully synchronized by coordinating the timing between the two systems to ensure that each measurement corresponded accurately to the probe's spatial position. Figure 2a illustrates the probe trajectory used during these measurements.

The measured SAR data were converted to 10 g‐averaged SAR by averaging over a volume equivalent to a 10 g mass of PVP. At 447 MHz, SAR validation was conducted to evaluate the spatial distribution of 10 g‐averaged SAR centered around the excitation ports of the bumped dipole elements for four different excitation patterns: circularly polarized (CP), uniform and two random‐phase excitations. Supplementary Table 1 lists the relative phase distribution with respect to Channel 1 for each excitation pattern. For this study, measurements were recorded across four axial planes centered at the coil's isocenter marker covering a circular area with a 59.5 mm radius. Total acquisition time was 48 minutes. Meanwhile at 297 MHz, SAR analysis of the 7T Nova 1Tx head coil was conducted across three volumetric reconstructions in axial, coronal and sagittal planes to verify the accuracy of the coil simulation prior to its use in a separate cranial mesh implant safety study.^38^ The same phantom and probe trajectory were used. The scanning depth was extended to 98 mm to allow for reconstruction across axial, coronal and sagittal planes. In total, fourteen axial planes were scanned across the phantom volume resulting in a total acquisition time of 168 minutes.

For both studies, the experimentally derived 10 g‐averaged SAR distributions were compared with electromagnetic simulations performed in CST Studio Suite (Dassault Systèmes, Darmstadt, Germany). The phantom geometry and electrical properties were modeled to match the experimental setup used in this work, while the coil model and simulation parameters followed the configurations described in the preceding studies.^4^, ^39^ The 10 g‐averaged SAR matrices (Q‐matrices), derived from EM simulations, were used to generate simulated SAR maps by applying the complex excitation vector corresponding to each experimentally examined excitation pattern.^40^ The numerically computed 10 g‐averaged SAR maps were then quantitatively compared with the experimentally acquired maps using the normalized root‐mean‐square error (NRMSE). This comparison was performed for each of the four excitation patterns of the 8‐channel head coil and for the three orthogonal planes (axial, sagittal, and coronal) of the Nova head coil.

### 2.3 Transfer function‐based safety assessment of a deep brain stimulation implant at 128 MHz

Safety assessment of RF‐induced heating around a directional deep brain stimulation (DBS) electrode (model 6173, Abbott Laboratories, Chicago, IL) was performed at 128 MHz using transfer function (TF) analysis.^41^ This formalism is widely used to characterize how incident electric fields couple to conductive implants and induce local heating. Missoffe et al introduced an experimental method to determine the TF of an elongated conductor by measuring the transmission coefficient along its length.^37^ In this work, the same method was applied to measure the TF of a DBS electrode terminated with a manufacturer‐provided silicone cap. TF measurements were conducted using a 1D automated linear motion system mounted on an acrylic phantom, controlled by a Raspberry Pi microcontroller (Cambridge, UK) connected to the computer workstation. The phantom is filled with PVP mixture (σ = 0.7 S/m, ε = 58 at 64 MHz, and σ = 0.74 S/m, ε = 55 at 128 MHz).^37^, ^41^, ^42^, ^43^ The electrode was suspended 6 cm above the phantom floor using plastic holders that were carefully positioned to avoid contact with the electrical contacts and metallic connection points on the electrode.

**FIGURE 3:**
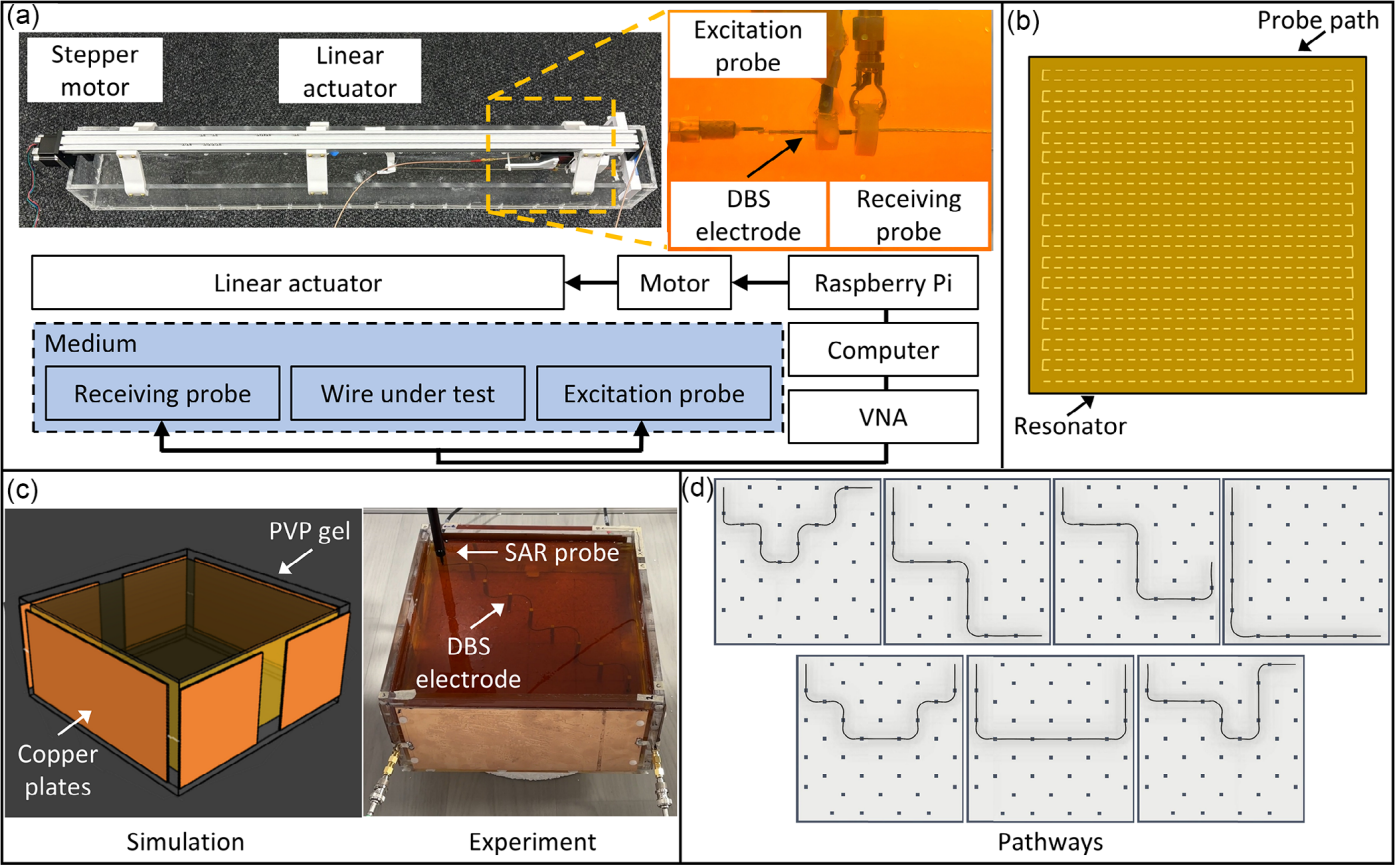
Setup for transfer function‐based safety assessments of a DBS electrode. (a) 1D linear motion system used to measure the transfer function of the DBS electrode with a computer workstation connected to the Raspberry Pi for motion control, and to a vector network analyzer for S‐parameter measurement. (b) Probe trajectory used for 10 g‐averaged SAR comparison for simulation model validation. (c) The SAR probe was positioned 5 mm away from the second most distal contact and recorded the SAR measurements for seven different electrode pathways (d).

An excitation monopole probe, made from a coaxial cable with its center conductor exposed, was placed in close contact with the most distal contact of the DBS. A receiving toroidal probe was positioned around the electrode and translated along its length in 10 mm increments to measure S12 parameter using a vector network analyzer (ZNL3, Rohde & Schwarz, Munich, Germany). S11 and S22 parameters were monitored throughout to ensure measurement stability and detect any potential deviations. Full two‐port calibration (through, open, short, and match) was performed at the SMA connectors closest to the monopole and toroidal probes prior to measurement at both measurement frequencies. The setup is shown in Figure 3a.

The accuracy and robustness of the measured TF must be validated to ensure reliable prediction of RF‐induced heating. Wang et al proposed a validation method in which active implantable medical devices (AIMDs) are exposed to the electric field generated by a rectangular resonator across multiple, highly orthogonal trajectories, and the temperature rise at the implant tip is compared with TF‐based predictions.^44^ The relationship between the TF and the predicted local SAR is given by:^28^, ^41^, ^42^, ^43^, ^44^

$$SAR=\alpha {\left|\int_{0}^{L}TF\left(l\right)E_{tan}\left(l\right)dl\right|}^{2}$$

where TF(l) is the spatially varying transfer function of the DBS and $E_{tan}\left(l\right)$ is the tangential component of the simulated electric field along the electrode length L.

In this study, the validation framework was adopted by using a rectangular resonator (25 × 25 × 12 cm^3^). Pointwise SAR was measured using the SAR probe for seven trajectories similar to what was used in the previous study. Because EM simulation is used to determine the incident *E_tan_* for each trajectory, the simulation model of the rectangular resonator needs to be validated. For this purpose, total input power of 75 W was supplied to the resonator. Pointwise SAR was measured across three axial planes and averaged to get 10 g‐averaged SAR. A rectangular resonator was filled with the same PVP mixture, and SAR was measured at the same vertical depth of 5 cm from the phantom floor. The measurement resolution was 10 mm, and the probe trajectory is displayed in Figure 3b. The total acquisition time for the scan was 82.6 minutes at each frequency. The resulting 10 g‐averaged SAR distribution was compared against simulated SAR from Sim4Life (ZMR Zurich MedTech, Zurich, Switzerland) to validate the electric field simulation model used in the TF‐based SAR predictions.

The validated simulation model of the resonator was used to extract the incident *E_tan_* along DBS electrode trajectories for TF‐based SAR predictions. For the TF validation experiments, SAR measurement was performed by positioning the SAR probe tip 5 mm from the second most distal contact of the DBS electrode. The electrode was placed along seven highly orthogonal trajectories (Figure 3c), and each pathway was subjected to 15 seconds of RF exposure. These measurements were used to compute the scalar calibration factor, for SAR prediction. By using the aforementioned SAR equation, α calculated as the mean ratio between the measured SAR and the calculated ${|\int_{0}^{L}TF\left(l\right)E_{tan}\left(l\right)dl|}^{2}$ was determined across all pathways. Because SAR levels may vary substantially among pathways, a lower total input power of 30 W was used to provide safe margin to prevent damage in cases of unexpectedly high coupling that causes SAR measurements exceeding the dynamic range of the probe. The predicted SAR was compared against the SAR probe measurements to assess the accuracy of the TF‐based SAR estimation.

## 3 RESULTS

### 3.1 RF coil characterization via SAR validation at 447 and 297 MHz

**FIGURE 4:**
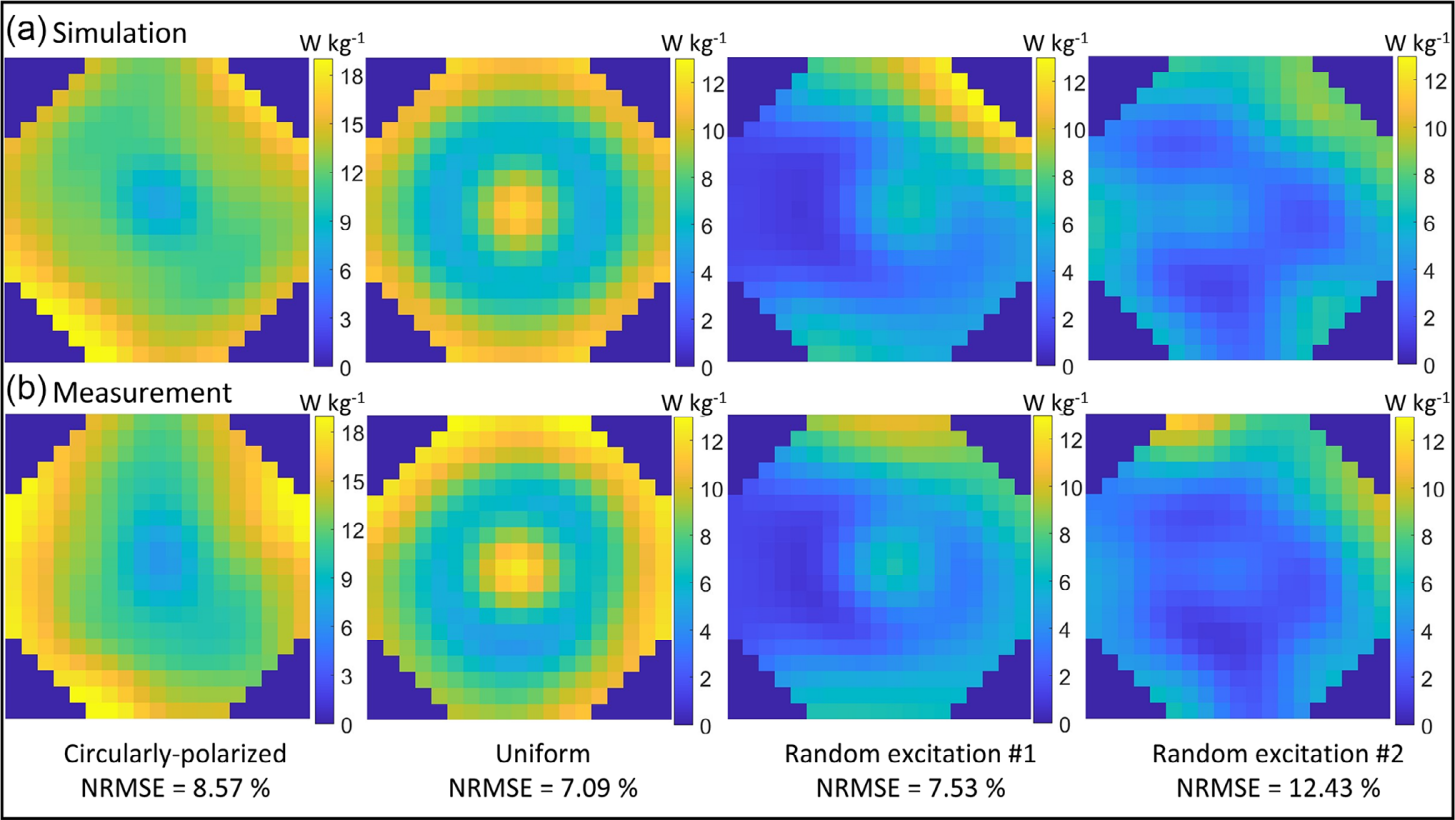
Comparison of simulated and measured 10 g‐averaged SAR distribution for four different excitation patterns with NRMSE < 13%.

To evaluate the linearity of the SAR probe and the transmit system, SAR was measured at two locations near one of the dipole elements of the bumped dipole head coil as the input power was incrementally increased from 1.5 W to 56.5 W. As shown in Supplementary Figure S1, the SAR measurement demonstrated linear behavior with respect to the total input power to the coil. Multiplane 3D SAR maps were acquired for four excitation patterns (circularly polarized, uniform and random), and 10 g‐ averaged SAR distributions were calculated. Figure 4 presents the simulated and measured 10 g‐averaged SAR maps for these excitation patterns, showing good agreement with less than 13% NRMSE. Notably, for the uniform excitation, interference patterns were observed not only at the periphery of the phantom near the individual dipole elements, but also in the central region of the phantom. This result highlights the importance of capturing the distribution of SAR in comparison to evaluating it at discrete measurement points with fiber optic temperature probes.

**FIGURE 5:**
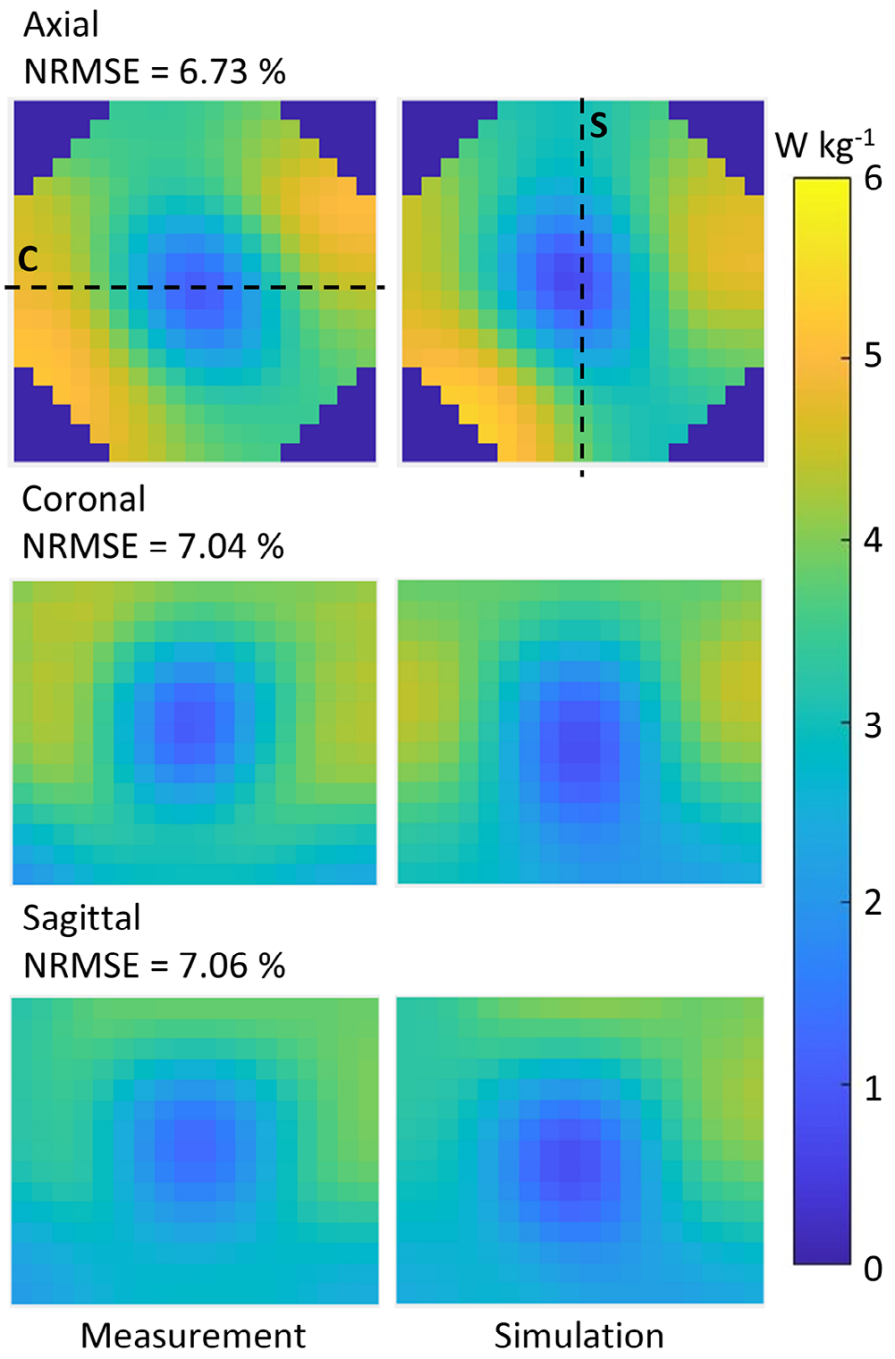
Results of Nova 1Tx coil characterization using volumetric 10 g‐averaged SAR comparison. (a) Experimental setup with a polyvinylpyrrolidone phantom centered within the transmit coil. The SAR probe trajectory was extended to cover 14 axial slices. (b) Axial, coronal, and sagittal comparisons of 10 g‐averaged SAR distributions between measurement and simulation, with NRMSE ≤ 10%.

As shown in Figure 5, the volumetric SAR acquisition allowed 10 g‐averaged SAR to be reconstructed in axial, coronal and sagittal planes, which offer comprehensive information about the spatial distribution of SAR. The axial planes covered a circular cross section with a radius of 59.5 mm while the coronal and sagittal planes spanned 119 × 98 mm^2^. At 297 MHz, the SAR measurements of the commercial 1Tx head coil showed agreement with simulation with NRMSE less than 8%.

### 3.2 Transfer function‐based safety assessment of a deep brain stimulation electrode at 128 and 64 MHz

**FIGURE 6:**
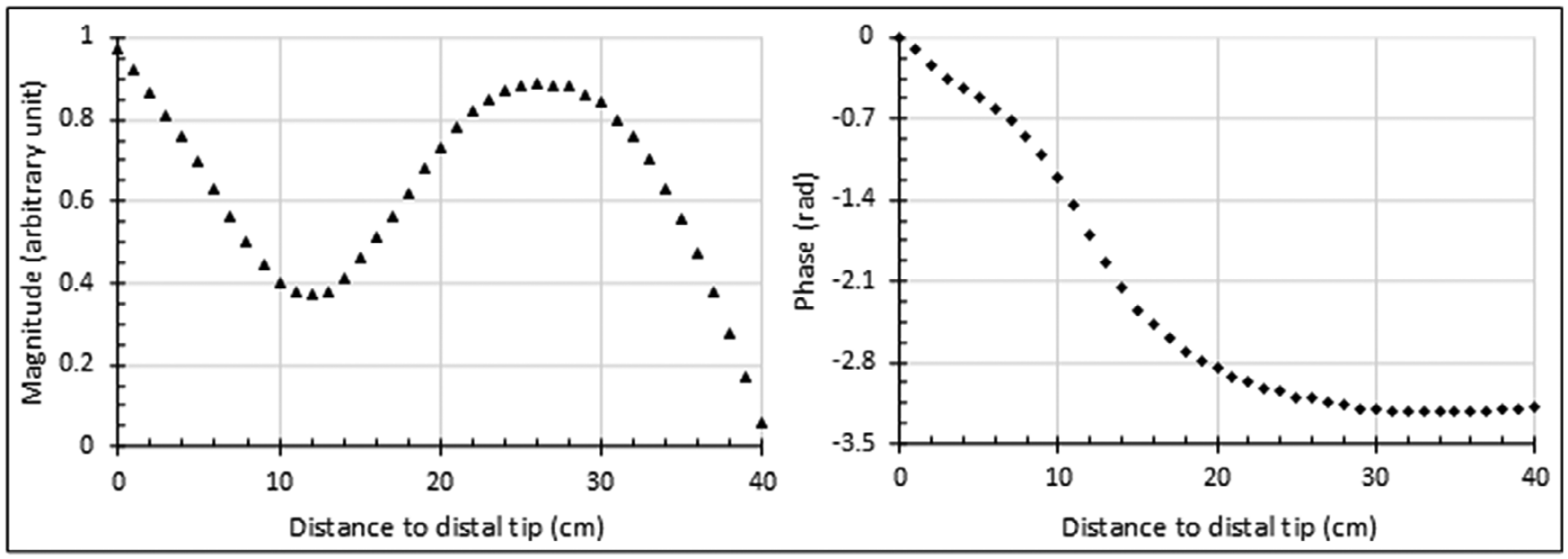
Transfer function measurement at 128 MHz of the DBS electrode with silicone cap acquired using the 1D automated linear system.

**FIGURE 7:**
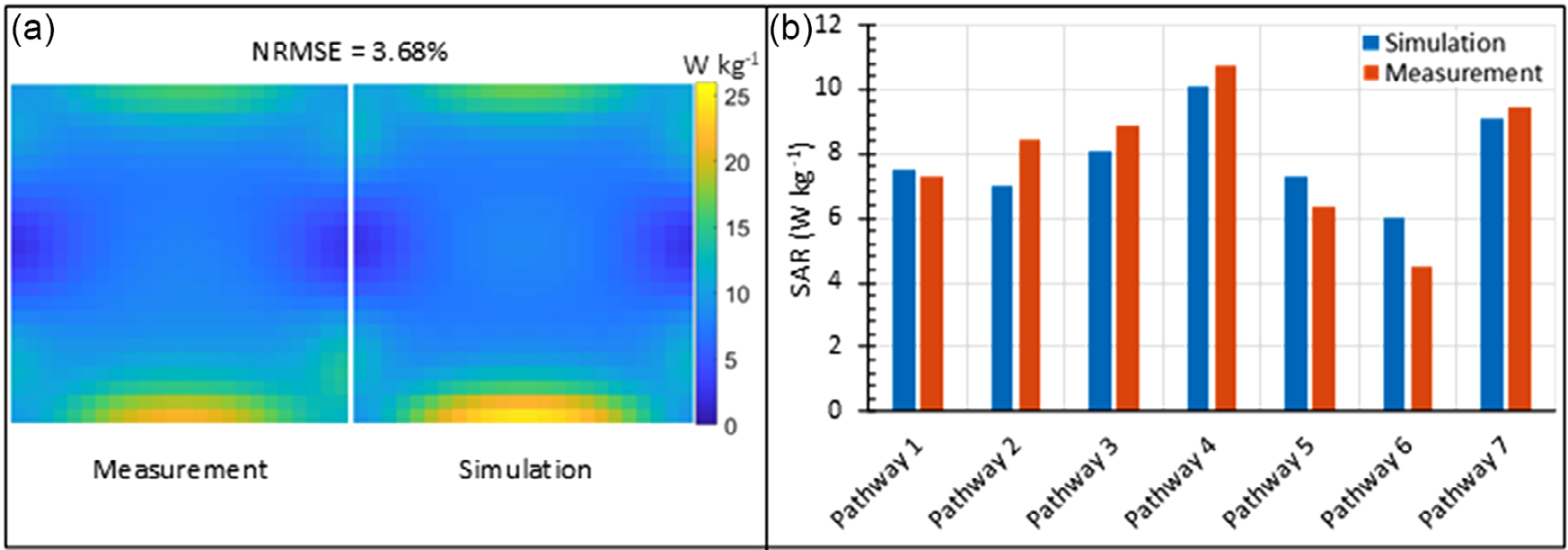
Results of the transfer function‐based safety assessment of a DBS electrode terminated with a silicone cap in a rectangular resonator with a polyvinylpyrrolidone gel (σ = 0.76 S/m, ε = 54). (a) Comparison of simulated and measured 10 g‐averaged SAR to validate the rectangular resonator model. (b) Comparison of transfer function–predicted and measured pointwise SAR following a 15 second RF exposure.

Figure 6 shows the measured transfer function of the directional DBS electrode at 128 MHz, acquired using the 1D automated linear system described in Section 2.3. It shows agreement with previously published data.^45^ Figure 7a compares the 2D distribution of 10 g‐averaged SAR measurements with simulated SAR distributions of the rectangular resonator used in the subsequent TF validation experiments at 128 MHz. The agreement between simulation and measurement with less than 4% NRMSE confirms the accuracy of EM model and simulation for extracting the tangential electric field information to predict SAR near the DBS electrode contacts. Figure 7b shows the comparison between predicted and measured SAR at the second most distal electrical contact of the DBS, using transfer function‐based SAR predictions and a SAR probe. The observed agreement with overall RMSE of 0.94 W/kg confirms the validity of the transfer function measured and supports the feasibility of using the SAR probe for this purpose.

## 4 DISCUSSION

In this work, we demonstrated the use of direct specific absorption rate (SAR) measurements in RF safety experiments. All experiments were done in a dedicated RF safety lab designed for performing safety validation studies in a controlled, B_0_ field‐free environment. The proposed approach offers several advantages over traditional MRI‐based safety validation workflows. First, the safety lab can support experiments across multiple field strengths, with the primary limitation being the bandwidth of the RF amplifiers. In our setup, we successfully generated RF excitations at 128, 297, and 447 MHz, covering a range of clinical and research‐relevant frequencies. With more advanced broadband amplifiers, even broader frequency coverage could be achieved to extend the applicability of the system. Second, the operational cost of experiments in the RF safety lab is minimal compared to scanner‐based studies. The need for spatially encoded imaging is also eliminated, therefore avoiding challenges associated with gradient non‐linearities and B_0_ inhomogeneity. Additionally, the use of non‐magnetic tools and equipment, which are often difficult to source for ultra‐high field systems, is no longer required. This advantage not only reduces the risk of projectile‐related accidents but also simplifies experimental workflows and troubleshooting processes. For example, standard diagnostic equipment such as an oscilloscope or VNA can be freely used to probe any part of the RF system, something that is typically impractical or unsafe within an MRI suite. This flexibility also supports high‐power testing and failure analysis of RF coils by safely causing failure modes such as RF arcing, followed by iterative evaluation of mitigation strategies to verify their effectiveness (see Supplemental Information and Fig S2). These advantages make a dedicated RF safety lab a highly efficient environment for addressing a wide range of RF safety challenges in MRI research, particularly for this form of simulation‐based validation of RF coils and implants, without requiring the full complexity and level of verification typically associated with regulatory evaluation workflows.

Direct SAR validation in B_0_ field‐free environments was demonstrated using a high‐precision SAR probe, calibrated to ISO/IEC 17025 standard, for both custom‐built and commercial RF coil arrays. A previous study used a home‐built E‐field probe to verify the electric field distributions from a multi‐channel RF heating device against simulation.^46^ Building upon this concept, probe‐based validation was extended to include direct SAR measurement to compare both spatial distributions and quantitative agreement between simulation and measurements. Several challenges commonly associated with heating‐based validation methods were avoided through this approach. Long cool‐off times are often necessary to reach a thermal steady state, increasing the duration of experiments to several hours.^24^ In addition, high RF power levels (average power > 80 W) are often required to generate measurable heating, which can exceed the operational limits of coil components, particularly in arrays with integrated electronics. In contrast, direct SAR measurements require less power to achieve sufficient SNR. For example, with the bumped dipole coil, measurements in low‐SAR regions were achieved as little as 17 W.^36^ Direct SAR measurements conducted in a B0 field‐free environment also avoid negative effects of phase‐related inhomogeneities and systemic errors due to non‐linear relationship between SAR and temperature, especially when heating curves have shallow slopes.

At 447 MHz, an 8‐channel transmit/receive bumped dipole head coil was characterized under multiple excitation conditions, and SAR distribution and system linearity were evaluated in a controlled manner. In contrast to prior work based on discrete thermometry, this study demonstrates direct SAR‐based validation of full central‐plane distributions across multiple excitation conditions, allowing direct comparison of spatial SAR distributions with EM simulations. This approach can be readily extended to volumetric validation given the automated measurement framework. At 297 MHz, this capability was further demonstrated through multi‐plane SAR mapping of a commercial Nova 1Tx head coil, where not only axial but also sagittal and coronal distributions were evaluated to validate the EM setup, which was subsequently used as the foundation for an implant safety study.^38^ Due to the automation in the measurement setup, including synchronized probe motion and acquisition through a centralized computer workstation, SAR measurements were completed within realistically short acquisition times, even with high spatial resolution and multiple excitation patterns. Further studies will explore the application of different probes in a range of safety evaluations beyond the scope of the current study.

Validation of the transfer function for a DBS electrode at 128 MHz was conducted using SAR probe measurements, demonstrating the lab's capability to support implant safety assessments. The pointwise SAR values measured with the probe showed strong agreement with the transfer function‐based predictions. As mentioned earlier, direct SAR measurements offer key advantages specifically in reducing the total experiment time since cool‐down period is not required like temperature‐based techniques. The SAR probe readings show negligible variation within a few minutes of exposure and even when RF power was toggled off and back on at the same power level. However, careful probe placement is important as the SAR probe has a defined dynamic range. To avoid exceeding this range, either the power level or the distance between the probe and SAR hot spots must be appropriately adjusted. The 3D motion system facilitated fine‐tuning the probe position during setup to ensure the probe is protected from exposure to excessive fields.

Beyond elongated devices like DBS electrodes, the RF safety of metallic implants with complex or three‐dimensional geometries such as titanium cranial meshes remains poorly understood particularly at ultra‐high field (UHF) strengths. For these devices, the location of peak SAR is difficult to predict, and temperature measurements using discrete sensors often fail to capture the true maximum. MR thermometry is further limited by B0‐related artifacts introduced by metal. The SAR measurement system demonstrated in this work enables high‐resolution 3D SAR mapping around such implants, overcoming limitations of conventional approaches. Moreover, the initial Nova coil characterization without the implant provides a validated baseline model of the transmit coil, forming the foundation for a subsequent study investigating cranial mesh safety in MRI.^38^ In that study, a titanium mesh was placed at the bottom of the phantom, and finer spatial resolution was used to reconstruct 1 g‐averaged SAR near the implant, validating also the implant model. Temperature rise measurements were also performed to verify the thermal simulations. Together, these three stages of coil validation, combined coil and implant SAR validation, and thermal validation, highlight the adaptability and rigor of direct SAR measurements in a B_0_ field‐free environment for verifying the accuracy of simulation models before introducing additional complexities such as varying implant orientation, geometry, and placement within different human models.

Future work will focus on extending the proposed validation framework to more complex RF configurations, including transmit/receive and receive array systems, where coupling effects and combined field interactions may further influence SAR distributions. The current multi‐plane acquisition approach can also be expanded to enable fully volumetric SAR validation, allowing more comprehensive comparisons with EM simulations. In addition, the application of this framework to implant safety can be extended to different implant geometries and operating frequencies to better characterize frequency‐dependent RF heating behavior, as demonstrated in prior work on a titanium mesh implant.^38^

## 5 CONCLUSION

This work demonstrates an experimentally grounded approach for direct validation of RF‐induced SAR, showing high agreement between simulated and measured results across multiple applications including coil‐based SAR assessment and transfer function‐based implant evaluation. This reliable framework is useful to strengthen confidence in the high use of EM simulation used for safety assessment. In addition to SAR validation, the B0‐free RF safety laboratory enables controlled testing of other safety‐relevant phenomena, where faults such as arcing can be systemically investigated. This environment supports effective troubleshooting and provides a platform for identifying coil‐related anomalies and assessing mitigation strategies. As MRI systems continue to evolve toward increasingly customized hardware, the ability to perform accurate and efficient safety assessments in a controlled, field‐free environment offers a significant advantage for research efforts.

## CONFLICT OF INTEREST STATEMENT

The authors declare no conflicts of interest.

## Supporting information



Supporting Information

Supporting Information

Supporting Information

Supporting Information
