# Supplementary material for: SAR validation of RF coils and implants in a B0‐free RF safety laboratory
Source: Med Phys. 2026 Aug 18;53(9):e70636. doi: 10.1002/mp.70636 (PMC13486907; doi:10.1002/mp.70636)
Supplement: Supplementary file 1 [file MP-53-0-s004.docx]

Supporting Information Table S1. Phase of each transmit channel relative to Channel 1 used in circularly-polarized (CP), uniform and two random excitations.

| Channel | CP | Uniform | Random #1 | Random #2 |
| --- | --- | --- | --- | --- |
| 1 | 0 | 0 | 0 | 0 |
| 2 | 45 | 0 | 25 | -58 |
| 3 | 90 | 0 | -55 | -88 |
| 4 | 135 | 0 | 24 | -37 |
| 5 | 180 | 0 | 23 | -1 |
| 6 | 225 | 0 | 26 | -56 |
| 7 | 270 | 0 | 32 | 7 |
| 8 | 315 | 0 | 3 | -99 |
