# Supplementary material for: SAR validation of RF coils and implants in a B0‐free RF safety laboratory
Source: Med Phys. 2026 Aug 18;53(9):e70636. doi: 10.1002/mp.70636 (PMC13486907; doi:10.1002/mp.70636)
Supplement: Supplementary file 2 [file MP-53-0-s002.doc]

# Supplementary Information

Supporting Information Table S1. Phase of each transmit channel relative to Channel 1 used in circularly-polarized (CP), uniform and two random excitations.

| Channel | CP | Uniform | Random #1 | Random #2 |
| --- | --- | --- | --- | --- |
| 1 | 0 | 0 | 0 | 0 |
| 2 | 45 | 0 | 25 | -58 |
| 3 | 90 | 0 | -55 | -88 |
| 4 | 135 | 0 | 24 | -37 |
| 5 | 180 | 0 | 23 | -1 |
| 6 | 225 | 0 | 26 | -56 |
| 7 | 270 | 0 | 32 | 7 |
| 8 | 315 | 0 | 3 | -99 |

*Supporting Information Figure S1. Linear response of the SAR probe with respect to the total input power of the bumped dipole head coil exposed to a circularly-polarized excitation. Measurements were performed at two positions, the center of phantom and the periphery.*

Testing under high-power conditions is an important step in evaluating the integrity of a custom-built transmit coil and its robustness under extreme operating condition [47]. A controlled failure test was conducted with a 16-channel transmit self-decoupled head coil in the RF safety lab [7] [44]. The coil was loaded with a head-shaped phantom filled with PVP (σ = 0.65 S/m and ε = 47.2). RF power was delivered to a single channel while terminating the remaining ones with 50 Ω terminations. An oscilloscope was used to detect anomalies during power delivery using the forward and reverse coupled ports of an in-line directional coupler.

Using a 0.5% duty cycle, RF power was gradually increased from 0 W while monitoring for visible signs of arcing or sparking. Once an event was successfully reproduced, an initial mitigation strategy was implemented on that channel. The procedure was repeated to confirm whether the applied solution effectively resolved the issue. Subsequently, other channels were examined under the same conditions to assess whether similar failures occurred, and the same mitigation strategy was systematically applied. In addition, a single-spot infrared (IR) thermometer was used to scan the surface of the coil and detect localized overheating or abnormal thermal signatures that could identify failure points.

Figure S2 shows the result for the high-power arcing test conducted with the 16-channel self-decoupled head coil at 447 MHz [7]. Arcing first occurred at a peak power level of 1200 W measured at the coil input. Initial arcing was localized at the sharp corners of the self-decoupled elements, as shown in Figure S2C. Oscilloscope traces in Figure S2B show sudden increases in reflected power during the RF pulse, coinciding with the arcing events. Subsequent testing produced arcing at lower power levels due to carbon build-up from the previous failures. To test the hypothesis that these corners were acting as sites of field concentration, the affected corner was cut at an angle as shown in Figure S2C. This modification successfully eliminated arcing failures. After applying the same angled-cut modification to all sharp corners across the array, no further arcing was observed, even at maximum output power of 2 kW achievable with the RFPA system.

These results demonstrate that the RF safety laboratory provides a controlled and repeatable environment for systematically reproducing and analyzing failure mechanisms under high-power conditions. Such effects are difficult to investigate within an MRI scanner, where faults may be intermittent and not readily detectable by system monitoring. In contrast, the safety lab enables precise RF power control, use of standard diagnostic tools, and repeatable testing, facilitating identification of coil-related anomalies and evaluation of mitigation strategies.

*Supporting Information Figure S2. Arcing event that occurred during high power delivery to a 16-channel self-decoupled RF coil array. A) Arcing was localized to one of the corners of an active channel. B) A disturbance in the reflected signal measured on an oscilloscope that coincided with the arcing event. C) The corners of the conductors were cut to remediate the arcing issue. Notice the burn marks (red arrows) around the vertices where arcing occurred compared to the clean vertices of a non-arced corner.*
